# Artisanal gold mining in Kakamega and Vihiga counties, Kenya: potential human exposure and health risk

**DOI:** 10.1007/s10653-023-01647-z

**Published:** 2023-06-20

**Authors:** Maureene Auma Ondayo, Michael J. Watts, Elliott M. Hamilton, Clive Mitchell, Joseph Mankelow, Odipo Osano

**Affiliations:** 1 Department of Environmental Health and Biology, University of Eldoret, P.O Box 1125, Eldoret, Kenya; 2 Inorganic Geochemistry, Centre for Environmental Geochemistry, British Geological Survey, Nottingham, NG12 5GG UK; 3 Mineral Resource Security and Flows, British Geological Survey, Nottingham, NG12 5GG UK

**Keywords:** Arsenic, ASGM, Bioaccessibility, Cancer and non-cancer health risks, Chromium, Kenya, Mercury, Risk assessment

## Abstract

Artisanal and small-scale gold mining (ASGM) represents 20% of gold supply and 90% of gold mining workforce globally, which operates in highly informal setups. Pollutants from mined ores and chemicals introduced during gold processing pose occupational and inadvertent health risks to the extent that has not been well elucidated in Africa. Trace and major elements were analysed using inductively coupled plasma mass spectrometry in soil, sediment and water samples from 19 ASGM villages in Kakamega and Vihiga counties. Associated health risks for residents and ASGM workers were assessed. This paper focuses on As, Cd, Cr, Hg, Ni and Pb for which 96% of soil samples from mining and ore processing sites had As concentrations up to 7937 times higher than the US EPA 12 mg kg^−1^ standard for residential soils. Soil Cr, Hg and Ni concentrations in 98%, 49% and 68% of the samples exceeded respective USEPA and CCME standards, with 1–72% bioaccessibility. Twenty-five percentage of community drinking water sources were higher than the WHO 10 µg L^−1^ drinking water guideline. Pollution indices indicated significant enrichment and pollution of soils, sediment and water in decreasing order of As > Cr > Hg > Ni > Pb > Cd. The study revealed increased risks of non-cancer health effects (98.6) and cancer in adults (4.93 × 10^−2^) and children (1.75 × 10^−1^). The findings will help environment managers and public health authorities better understand the potential health risks in ASGM and support evidence-based interventions in ASGM processes, industrial hygiene and formulation of public health policy to protect residents and ASGM workers’ health in Kenya.

**Supplementary Information:**

The online version contains supplementary material available at 10.1007/s10653-023-01647-z.

## Introduction

With the increasing value of gold and difficulty earning a livelihood from agriculture and other rural income-generating activities, the artisanal and small-scale gold mining (ASGM) sector has rapidly grown over the past 50 years in low- and middle-income countries (Schwartz et al., 2021; World Bank, 2009). In 1999, for instance, an estimated two million people directly engaged in ASGM activities worldwide, up to 10 million in 2019 and over 20 million supporting the livelihoods of 100 million people in 2022 (Fritz et al., 2018; Radley, 2022; World Gold Council, 2022). This rapid expansion of ASGM activities has resulted in increasing global public health concerns about the life-threatening toxic pollution from the sector. Typical ASGM activities are low capital, are poverty driven, use crude technology and are often conducted in remote villages where environmental, health and safety practices and regulations are unsurprisingly either recently evolving, not stringent or lacking (Fritz et al., 2018; Plumlee et al., 2013; Rajaee et al., 2015; Schwartz et al., 2021). As a result, large amounts of potentially toxic elements (PTEs)-contaminated dust, vapours and solid and liquid wastes from ASGM operations are usually released into nearby soils, water sources, food sources and air unabated. These pose severe health risks to the ASGM workers and nearby communities, and associated fatalities have been documented globally (Dooyema et al., 2012; N'goran et al., 2021; Odukoya et al., 2021; Plumlee et al., 2013; Rajaee et al., 2015). In Africa, most of the risks posed by ASGM are borne by women and children (Fritz et al., 2018; Steckling et al., 2014, 2017). It is also estimated that over 9 million ASGM workers support over 150 million people across Africa (Fritz et al., 2018; Hilson, 2016; Jønsson & Fold, 2009; Schwartz et al., 2021; World Bank, 2009).

Artisanal and small-scale gold mining (ASGM) accounts for 20% of the gold supply and 90% of the gold mining workforce globally, which operates in highly informal setups (World Gold Council, 2022). It is estimated that 60% (3.6 tons) of Kenya’s annual gold production originates from the ASGM sector, which supports over 250,000 ASGM workers and ~ 1 million dependent family members with livelihoods (Barreto et al., 2018; planetGold, 2021). The promulgation of the new mining act in Kenya, Mining Act No. 12 of 2016 (section 98, subsections 2 and 3) formalised and legalised ASGM activities countrywide, allowing for licensing and putting in place a framework for best practices in a sector that was largely considered illegal and informal (Fritz et al., 2018; GoK, 2016). However, the act lacks specific regulations on the health and safety of ASGM workers and local communities. Furthermore, apart from the Minamata Convention on Mercury (Minamata Convention on Mercury, 2021) Kenya has not ratified many International Labour Organisation (ILO) conventions on health and safety, including the Safety and Health in Mines Convention, 1995 (No. 176) (ILO, 2023).

The predominant ASGM activities of ore excavation, crushing, milling, gravity separation on sluices and mercury amalgamation and subsequent vaporisation may result in human exposures to PTEs released from ores or chemicals applied in gold beneficiation such as mercury (Hg) and sodium cyanide (NaCN) (Basu et al., 2011; Dooyema et al., 2012; Plumlee et al., 2013; Rajaee et al., 2015; Tomicic et al., 2011). Elsewhere in Africa, catastrophic outcomes have been reported from high concentrations of Pb (175,000 mg kg^−1^) that was introduced into the environmental media (soil, dust, water and food) (Dooyema et al., 2012; Plumlee et al., 2013). In Kenya, the World Health Organization (WHO) guidelines for As, Cd, Cr, Hg, Ni, Pb and other toxic elements in soil, sediment and water from ASGM areas in the Migori Gold Belt, Transmara, and Lake Victoria were reported to have been exceeded (Mutono, 2016; Ngure et al., 2014; Odumo et al., 2011, 2018; Ogola et al., 2002; Okang’Odumo et al., 2014). However, studies that quantify pollution and determine potential human exposures and health risks associated with these PTEs are limited. The new mining laws and regulations provided opportunities to conduct investigations and offer evidence-based measures for sustainable ASGM activities (GoK, 2016). Therefore, this study aimed to determine the environmental and potential public health consequences of ASGM activities in Kakamega and Vihiga counties in western Kenya as guided by the following objectives: (1) environmental characterisation of PTE concentrations in soil, ores, mine wastes, sediments and waters within ASGM villages in Kakamega and Vihiga counties, Kenya, and (2) calculate associated cancer and non-cancer health risks to ASGM workers and the local communities.

## Materials and methods

### Study area

**Fig. 1:**
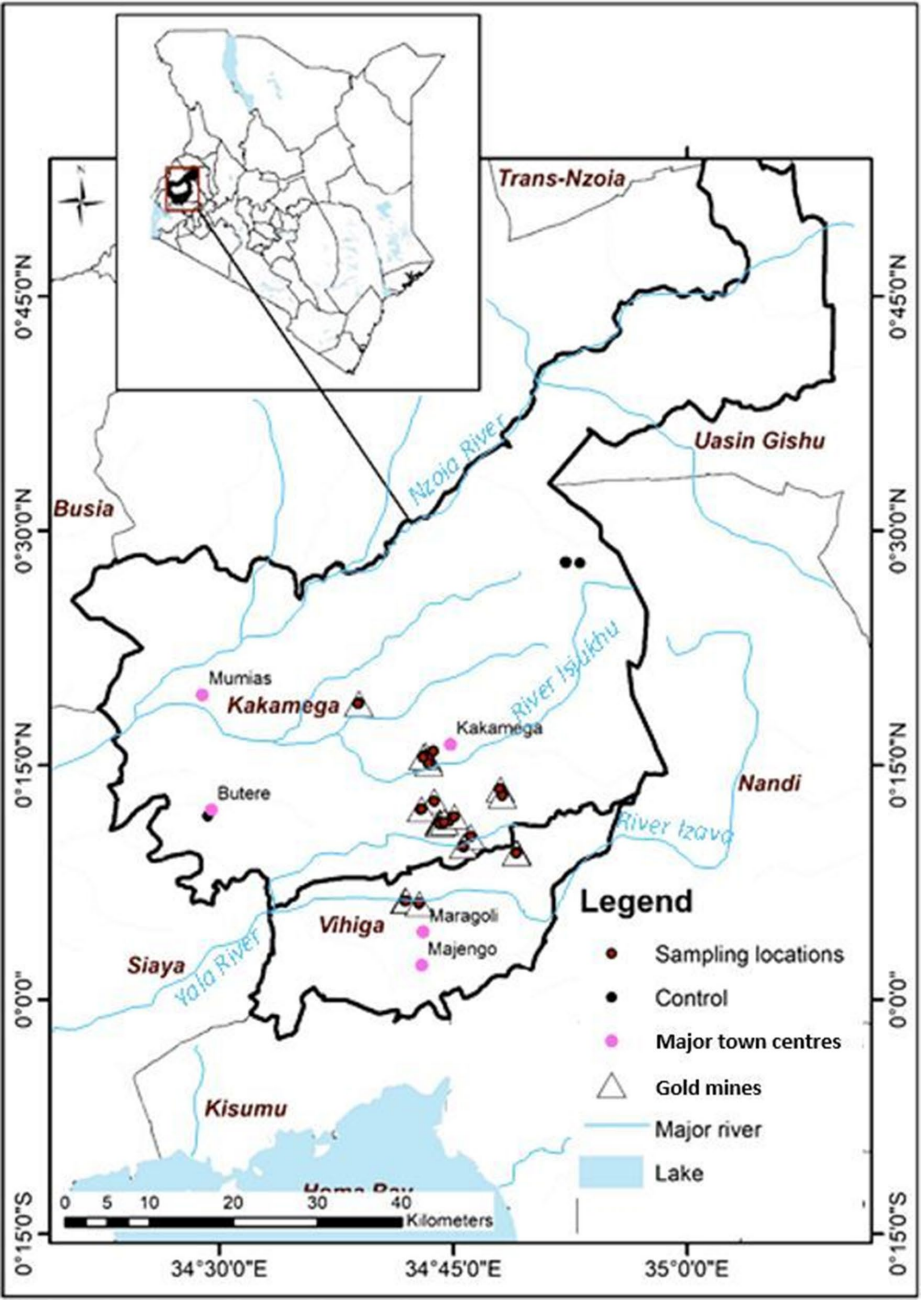
Map showing sampling sites in Kakamega and Vihiga counties, Kenya

Located in western Kenya, Kakamega and Vihiga counties have populations of 1,867,579 and 590,013, respectively, with gross incomes of US $1910 and US $1848 per capita, respectively. They are considered among the most densely populated counties in Kenya: Kakamega; 618 people km^2^ and Vihiga; 1047 people km^2^ with poverty rates of 57% and 62%, respectively. The major underlying burdens include food insecurity, poor healthcare infrastructure, waterborne diseases, malaria, HIV/AIDS and inadequate drinking water among others (KNBS, 2019). Kakamega and Vihiga counties lie within altitudes of 1240–2000 m and 1300–1640 m above sea level, respectively (Fig. 1). Both counties are well drained with permanent rivers such as Isiukhu, Izava, Yala and Nzoia, which drain their waters into Lake Victoria and are the primary water sources for drinking, domestic use and supplying fishponds and irrigation. The mean annual rainfall ranges from 2000 to 2200 mm in Kakamega county and 1800 to 2000 mm in Vihiga county and is bimodal with long (March–July) and short (September–October) rainy seasons (Humphrey et al., 2022). The geology of Vihiga and Kakamega counties has the formation of the Kavirondian and the Nyanzian volcanic systems. The Nyanzian system is mainly gold-bearing auriferous quartz veins from underlying geology (County Government of Vihiga, 2014; Huddleston, 1954). Kakamega county has two main ecological zones. First is the Upper Medium (Central and Northern parts), where intensive maize, beans and horticultural production is done. Second is the Lower Medium zone, which mainly covers most of the southern part of the county with sugarcane as the main cash crop in addition to subsistence production of maize, sweet potatoes, cassava, groundnuts and tea. The natural tropical rain forest covers an estimated 244 km^2^ area, while planted forests are integrated with agricultural farming covering an area of about 26.5 km^2^ (County Government of Kakamega, 2018). But, in Vihiga county, much of the natural vegetation was replaced by human settlement and subsistence farming. The major crops grown are sugar cane, maize, bananas, arrowroots, beans, vegetables and Napier grass. Natural tropical rainforests cover 37 km^2^ and planted forests cover approximately 0.5 km^2^. Eucalyptus forms 70% of exotic trees and is planted along river valleys and in agricultural farms as income sources (County Government of Vihiga, 2014).

Multi-ethnic communities live and work as artisanal small-scale gold miners and ore processors, fishers and farmers. In addition, the local communities in both counties consume freshwater fish caught locally in the rivers Isiukhu, Izava, Yala and Nzoia and Lake Victoria, which are also used for irrigation as well as recreational sites for swimming. Traditionally, locally grown food is consumed, with many residents relying on subsistence farming. This ongoing cross-sectional study commenced in February 2020. Kakamega and Vihiga counties were chosen based on the historical mining of gold and identification as current ASGM hotspots that are expanding operations with little information on potential sources of environmental and human exposure to PTEs (Fig. 1).

Background study sites with similar geological characteristics, agricultural, industrial and traffic activities like those in the study area but with no recorded mining activity, were chosen as control sites in five villages in Kakamega (Butere, Tande, Lugusi, Shiatsala and Eshishebu villages) and one village in Eldoret (Elgon View Estate) (Fig. 1). Unique numbers were generated for all the ASGM sites and schools. From these, based on the possible PTEs migration routes such as wind direction, soil, water, sediment and potential downstream dispersion, among others in the study area, sampling points within 19 ASGM villages were selected using unique numbers and included in the study. A similar procedure was used to select and include background/control sampling points (three sites and two schools) in the study.

### Sample collection and preparation

Soil, sediment and water sampling protocols were based on the field collection reported in Watts et al. (2019). Using a clean plastic scoop, 121 composite topsoil samples (each made of five sub-samples collected within a 20-m^2^ plot) were collected 0–15 cm deep from sites referred to as: industrial, for areas where ASGM activities such as ore excavation/mining, crushing, grinding, washing and amalgam burning were conducted; ores, for piles of fresh ores from several feet underground; mine waste/tailings, referring to mixtures of ground ores and rocks which remain after recovering gold from typical ASGM setups or virgin ores; exhausted mine waste/tailings, representing heaps of exhausted/mine wastes/tailings from cyanidation establishments; residential, for homesteads and other places where people live in the villages; agricultural, from cultivated farms; children’s playgrounds; schools within the ASGM villages; and the control, from non-ASGM. Each soil sample was labelled appropriately and stored in a Kraft bag on site.

Sediment samples were collected from the ore washing ponds, fishponds, rivers, streams, springs and ponds where exhausted tailings or mine wastes from cyanidation/leaching plants are deposited. The top oxidised layer was removed using a pre-cleaned scoop; sediment samples were collected, labelled and stored in Ziploc plastic bags. The soil and sediment samples were freeze-dried, disaggregated and sieved to < 2 mm, at the Biotechnology Laboratory, University of Eldoret, Kenya. From the sieved < 2 mm sample, a 60-g fraction was drawn and stored in Kraft bags before transportation/export. Triplicate drinking and non-drinking water samples were collected from springs, rivers/streams, underground shafts, shallow wells, boreholes, washing ponds and fishponds. Two of the water samples (15 mL each) at each point were filtered on-site using 0.45-µm syringe filters, after which one sample was acidified with concentrated 1% v/v HNO_3_ (Sigma-Aldrich, Germany) and the third sample, 30 mL, was unfiltered and un-acidified. All the water samples were appropriately labelled and refrigerated. All prepared soil, sediment and water samples were thereafter packed and transported to the UK, for further preparation, digestion and elemental analysis. The activities, ASGM processes and population characteristics observed at each sampling location pointed to potential occupational, non-occupational or both exposures to PTEs.

### Sample dissolution and elemental analyses

Mercury concentrations in soil and sediment were determined using a direct mercury analyser (DMA-80, Milestone Inc., Italy) before oven-drying the samples. The soil samples were further oven-dried at 40 °C. A 0.25-g sub-sample of each dried sample was dissolved for a broad group of trace and major elements in a mixed acid solution (HF:2.5 mL/HNO_3_:2 mL/H_2_O_2_:2.5 mL) on a programmable hot block, heated in a 15-mL Nalgene HDPE bottle at 70 °C in a drying oven for 3 h and then diluted with 5 mL of deionised water followed by centrifugation at 3000 rpm for 15 min, from which the digest was used for analysis as described by Watts et al. (2019). To each acidified water sample, 0.5% v/v HCl and 1% v/v HNO_3_ were added. Total metal concentrations were determined using inductively coupled plasma mass spectrometry (ICP-MS) (Agilent 8900 ICP-QQQ). The detection limits were calculated as 3 × SD of blanks × dilution factor and presented with the data to evidence the uncertainty on measurements for soil (Supplementary Table 1a), sediment (Supplementary Table 3) and water (Supplementary Table 5). Bioaccessible elemental extractions and analyses were done for selected soils (Hamilton et al., 2015). In addition, mineralogical characterisation was carried out on 5 g of each of the milled sub-samples of the ore by X-ray diffraction (XRD) analysis using a PANalytical X’Pert Pro series diffractometer. Diffraction data were analysed using PANalytical X’Pert HighScore Plus version 4.9 software coupled to the latest version (at time of analysis) of the International Centre for Diffraction Data (ICDD) database.

### Quality control and assurance

Detailed standard procedures for collecting, transporting, storing and analysing samples were followed (Johnson & Breward, 2004; Watts et al., 2019). Analytical super purity grade chemicals (Romil, UK) were used throughout sample digestion and analyses. Deionised water with a resistivity of 18.2 MΩ (Millipore, UK) was used throughout digestion and analysis. Certified reference materials (CRM): 2711a Montana Soil (National Institute of Standards and Technology (NIST), BCR-2 Basalt Soil, BGS 102 Ironstone Soil (British Geological Survey), MESS-4 Sediment (Marine Sediment CRM for Trace Elements and other constituents) and SLRS6 Natural water (National Research Council of Canada (NRC–CNRC), were used to validate the dissolution and analytical procedures—all soil, sediment and water data are reported with the certified reference material data (Supplementary Tables 1, 3 and 5). Analysis of Hg was not done on the water samples due to the volatile nature of Hg and lack of an appropriate sample collection method to trap it before escaping—this will be addressed in follow-up work.

### Typical artisanal small-scale gold mining as practised in Kakamega and Vihiga counties

In the Kenyan counties of Kakamega, Vihiga, Siaya and Migori, there is a legacy of ~ 90 years history of ASGM. Using crude tools and methods, the miners explore alluvial gold deposits in about 5% of operations (Supplementary Table 12, Photographs 1a and 1b) or generally excavate between 300 and 500 m (Supplementary Table 12, Photograph 2) or use explosives to bring to the surface PTEs-contaminated ores for processing. The first processing stage involves crushing the ores using sledge and mason’s hammers (Supplementary Table 12, Photograph 3a). After crushing, the ore is sun-dried (Supplementary Table 12, Photograph 3b) and ground using locally fabricated gasoline-powered ball mills into fine ore powder, within residential villages, farms and waterways. Large amounts of dust are released during the mechanised grinding (Supplementary Table 12, Photograph 4). The ground ore is then wetted and passed through sluices to concentrate gold particles. The sluices are locally made wooden boxes covered with cloths that utilise gravity concentration to separate gold from the ores, gravel and dirt (Supplementary Table 12, Photographs 5 and 6). Finally, the sluice cloths containing concentrated gold are washed in water basins and buckets. Panning by swirling in a basin is thereafter done to further re-concentrate the gold obtained from sluicing. The process utilises a metal pan and water to separate gold from soil, rocks and gravel. Wet concentrate from sluicing is placed on the pan, wetted and stirred in circular motions using bare hands (Supplementary Table 12, Photograph 7a). This procedure breaks the lumps present. The pan is then rotated spirally and tilted at intervals causing materials lighter than gold to spill out. Gold is left in the pan and separated easily (Supplementary Table 12, Photograph 7b). Afterwards, liquid mercury (Hg) is added and kneaded onto the concentrate (Supplementary Table 12, Photograph 7c). Mercury amalgamates with gold (Supplementary Table 12, Photograph 7d). The mercury–gold (Hg–Au) amalgam is heated using liquefied petroleum gas, charcoal stoves, jikos or other open flames to obtain the gold as Hg vaporises (Supplementary Table 12, Photographs 8a and 8b) This is usually done in gold shops locally known as *kiosks,* kitchens, living rooms as well as in the open. Gold is chiefly sold to standby gold-brokers. Management of wastes from the ASGM activities, including tailings, exhausted wastes from cyanidation plants and wastewater, is poor. Both the solid and liquid wastes are haphazardly disposed of in nearby soils in farms, homesteads, playgrounds and waterways (Supplementary Table 12, Photograph 9). Some ASGM workers store their ground ores and wastes and reprocess them inside their houses and family compounds. Furthermore, as observed, the ASGM miners and ore processors do not wear personal protective equipment while working (Supplementary Table 12, Photographs 1–9). The toxic elements associated with the ASGM operations are likely to find their way into drinking water sources, food chains, the ambient air and finally into human and animal systems if ingested, inhaled or via the skin (Supplementary Table 12, Photographs 7, 8 and 9).

In Kakamega and Vihiga counties, three mining methods were observed depending on the location of gold deposits. First, the commonly used surface mining methods include open cast and alluvial mining. Alluvial mining involves sifting out and recovering gold from topsoil, sediments in ore processing ponds, various streams and rivers Isiukhu, Izava and Yala channels. Secondly, hard rock mining uses shafts between 300 and 500 m deep to extract gold ores. In both scenarios, ore processing utilises fluid Hg to recover gold. The third mining method observed in the study area was mechanised cyanidation/hydrometallurgy. This method usually utilises an alkaline cyanide leaching process to recover gold, primarily NaCN, to oxidise gold-containing virgin ores and tailings and the separation of metallic gold from the solution by reductive precipitation.

### Pollution indices used to estimate the extent of PTEs pollution

#### Index of geoaccumulation

Index of geoaccumulation (Igeo) was used to estimate the enrichment of metal concentrations above the baseline or background concentrations given by the Muller equation (Eq. 1) as follows:

1 $${\text{Igeo}}\, = \,\log_{2} [{\text{Cm}}/1.5\;{\text{BmE}} ]$$

where *Cm* is the measured concentration of element *m* (As, Pb, Hg, Cr, Cd and Ni) (mg kg^−1^) in the soil, sediment and water sample and *Bm* is the background geochemical concentration of the element *m*. The constant 1.5 rectifies for the natural fluctuations between the content of a given metal in the environment and some anthropogenic influences. Six classes of increasing Igeo values exist as follows: 0 < Igeo ≤ 0 practically unpolluted; 0 < Igeo ≤ 1 unpolluted to moderately polluted; 1 < Igeo ≤ 2 moderately polluted; 2 < Igeo ≤ 3 moderately to heavily polluted; 3 < Igeo ≤ 4 heavily polluted; 4 < Igeo ≤ 5 heavily to extremely polluted; and Igeo ≥ 5 extremely polluted (Barbieri et al. 2016; Förstner & Müller, 1981; Odukoya et al., 2021; Watts et al., 2017). Background element concentrations for the study area are available (Watts et al., 2019), except for Hg in which case, geochemical concentrations obtained for the pristine/forest area and the control were used.

#### Enrichment factor

Enrichment factor (EF) is used to determine whether total elements found in higher concentrations than the baseline concentrations in the earth’s crust could be of anthropogenic origin (N'goran et al., 2021; Watts et al., 2017). It is usually calculated as a ratio of measured sample metal concentrations above the reference material concentrations in the earth’s crust as per the following formula:

2 $${\text{EF}}\, = \,(C_{{{\text{Metal}}}} /C_{{{\text{normaliser}}}} ){\text{ Sample}}/(C_{{{\text{Metal}}}} /C_{{{\text{normaliser}}}} ){\text{ Background}}$$

where (*C*_metal_/*N*_ormaliser_) sample is the ratio of respective concentrations of each metal (As, Pb, Hg, Cr, Cd or Ni) and the normalizer (in this case, Iron (Fe)) in the sample. (*C*_Metal_/*C*_normaliser_) background or control is each metal and Fe concentration ratio in the background/control. Iron (Fe) has been widely used as a normaliser. The contributions of a given element from an anthropogenic source increase as the EF increases. Based on EF, there are five contamination categories. Enrichment factor (EF) < 2 shows deficiency to minimal enrichment; 2 ≤ EF < 5 indicates moderate enrichment; 5 ≤ EF < 20 is significant enrichment; 20 ≤ EF < 40 means very high enrichment; and EF > 40 shows extremely high enrichment (Watts et al., 2017).

#### Single pollution index (PI) and Nemerow integrated pollution index (NIPI)

Single pollution index (PI) determines which element represents the highest threat. It is a ratio of content of the element in sample and the geochemical background, given by:

3 $${\text{PI}}\, = \,Cn/Bn$$

There are three PI classes, namely PI <  = 1, low contamination; 1 < PI <  = 3 , low contamination; and PI > 3, high contamination (Odukoya et al., 2021). From the PI values, the overall pollution of soil, sediment and water from all six elements was calculated using the Nemerow integrated pollution index (NIPI) as per: NIPI = [0.5 × (PI^2^_average_ + PI^2^_max_)]^1/2^, where PI_average_ and PI_max_ are the average highest of all pollution indices considered (Mahfooz et al., 2020).

### Bioaccessible fraction (BAF%)

The BAF% of As, Cd, Cr, Hg, Ni and Pb in ores and soils from mining and processing sites, residential and agricultural areas, schools and playgrounds was calculated as a percentage of the ratio of each element concentration in the stomach and intestine phase (the highest) to the total concentration as per Hamilton et al. (2015). The bioaccessible hazard quotient was then generated as a product of BAF% and respective hazard quotients (HQ) (Hamilton et al., 2015; Odukoya et al., 2021).

### Estimating potential human exposure and associated health risk

Risk is evaluated for both cancer (cancer) and non-cancer (non-cancer) health effects (Gerba, 2019; Means, 1989; WHO, 2010c). In this study, the potential cancer and non-cancer health risks caused as a result of ingestion, inhalation and dermal absorption of the PTEs in various soils; ingestion and dermal contact of PTEs in various waters; and dermal contact of PTEs in sediments in the studied ASGM villages in Kakamega and Vihiga counties were assessed after estimating the average daily doses (ADD), the hazard quotient (HQ) and the lifetime daily doses (LADD) for children and adults.

#### Average daily dose (ADD)

The average daily dose (ADD) (mg kg^−1^ day^−1^) for soil, sediment and water was estimated using Eqs. (4, 5, 6, 7) for children and adults. The exposure factors, abbreviations and interpretations and values of input parameters for computing the dose and risk for this study are summarised in Supplementary Table 7 as per the US EPA, Environmental Health Assessment Guidelines and include the field and laboratory data from this study as well. The average daily dose for ingestion of PTEs via soil and water (ADD_ing_) was calculated as:

4 $${\text{ADD}}_{{{\text{ing}}}} \, = \,c \, \times \, R_{{{\text{ing}}}} \times {\text{ EF }} \times {\text{ ED }} \times {\text{ CF}}/{\text{ BW }} \times {\text{ AT}}$$

The average daily dose for dermal absorption of PTEs particles via soil and sediment (ADD_derm_) was calculated as:

5 $${\text{ADD}}_{{{\text{derm}}}} \, = \,c \, \times {\text{ SA }} \times {\text{ FE }} \times {\text{ AF }} \times {\text{ ABS}} \times {\text{ EF }} \times {\text{ ED }} \times {\text{ CF}}/{\text{BW}}. \, \times {\text{ AT}}$$

The average daily dose for dermal absorption of PTEs particles via water (ADD_derm_) was calculated as:

6 $${\text{ADD}}_{{{\text{derm}}}} \, = \,c \times {\text{SA}} \times K_{p} \times {\text{ABS}} \times {\text{ET}} \times {\text{EF}} \times {\text{ED}} \times {\text{CF}}/{\text{BW}}. \times {\text{AT}}$$

The average daily dose for inhalation of PTEs particles via soils (ADD_inh_) was calculated as:

7 $${\text{ADD}}_{{{\text{inh}}}} \, = \,c \times {\text{R}}_{{{\text{inh}}}} \times {\text{EF}} \times {\text{ED}}/{\text{BW}} \times {\text{AT}} \times {\text{PEF}}$$

#### Non-cancer risk assessment

The magnitude of exposure potential or quantifiable probability of occurrence of non-cancer health effects as a result of ingestion, inhalation and dermal absorption of PTEs (As, Cd, Cr, Hg, Ni and Pb) in the various soils; ingestion and dermal contact of PTEs in drinking and non-drinking waters; and dermal contact of PTEs in sediments in children and adults after a given exposure period was evaluated using the hazard quotient (HQ). The HQ is calculated as a ratio of the average daily dose (ADD in mg kg^−1^ day^−1^) of the PTEs with the respective reference doses (RfD in mg kg^−1^ day^−1^) (Supplementary Table 8) (Gerba, 2019; Means, 1989; USEPA, 2004), as;

8 $${\text{HQ}}\, = \,{\text{ADD}}/{\text{RfD}}$$

Based on the threshold of RfD value, evaluating existing adverse human health effects to humans is possible. An RfD value higher than the ADD shows that there would be no any adverse health effects. HQ values lower than 1 indicate lower to no adverse health effects, while HQ values greater than 1 show likely adverse health effects (Gerba, 2019). The total health hazards per pathway, referred to as hazard index (HI), were calculated by summing the HQ values (Gerba, 2019; Means, 1989; USEPA, 2004) given as follows:

9 $${\text{HI}}\, = \,{\text{HQ}}_{{1}} \, + \,{\text{HQ}}_{{2}} \, + \,{\text{HQ}}_{{3}} \, + \,{\text{HQ}}_{4} \cdots \, + \,{\text{HQ}}$$

Hazard index (HI) gives the total non-cancer risk of every single pathway in a given mix. Values lower than 1 indicate no significant non-cancer health risk. Values of HI greater than 1 depict existing likelihood of non-cancer health effects occurring and the probability increases as the values rise. This study assessed non-cancer health risks as a result of exposure to multiple elements through various pathways, mainly ingestion, inhalation and dermal contact. The overall potential non-cancer health risks for all the possible pathways in the ASGM villages in Kakamega and Vihiga counties were therefore estimated as the total exposure Hazard Index (HIt), obtained by summing up all the HI values for ingestion (HIing), inhalation (HIinh) and dermal contact (HIdermal) (Eq. 10) (Gerba, 2019; Means, 1989; USEPA, 2004), given as follows:

10 $${\text{HIt}}\, = \,{\text{HIing}}\, + \,{\text{HIinh}}\, + \,{\text{HI dermal}}$$

#### Cancer risk assessment

Cancer risks show the probability of a given population developing any type of cancer as a result of intake of carcinogens. The cancer risks associated with the intake of As, Pb, Hg, Cr, Cd and Ni in soil, sediment and water were calculated based on the lifetime average daily dose (LADD), also referred to as the chronic daily intake (CDI), for ingestion, dermal and inhalation routes of exposure as well as their respective International Agency for Research on Cancer (IARC) classification (Gerba, 2019; IARC, 2021; Means, 1989). The respective LADD for As, Pb, Hg, Cr, Cd and Ni in soil, sediment and water via ingestion, inhalation and dermal pathways were generated using respective parameters in Supplementary Table 7 as per Eqs. (4), (5), (6) and (7). The incremental lifetime cancer risk (ILRC) was calculated for children and adults as a product of LADD and cancer slope given by:

11 $${\text{ILCR}}\, = \,{\text{LADD }}*{\text{ SF}}$$

The total ILCR for an individual within the studied ASGM villages was finally calculated from the mean contribution of individual elements for all the pathways as,

12 $${\text{Risk}}_{{\text{(total)}}} \, = \,{\text{Risk}}_{{\text{(ingestion)}}} \, + \,{\text{Risk}}_{{\text{(inhalation)}}} \, + \,{\text{Risk}}_{{\text{(dermal)}}}$$

The RfD and SF values used for calculating both cancer risk and non-cancer risk, respectively, are shown in Supplementary Table 8.

### Statistical analysis

The data were stored and analysed in Excel (Microsoft Corporation) and Minitab version 21.0 (Minitab Inc.). The Ryan–Joiner test showed that the element concentrations data in soil, sediment and water were positively skewed; thus, the geometric means were calculated in addition to arithmetic means. Data were natural log (ln)-transformed prior to statistical analyses. The range, percentiles, geometric mean and arithmetic means were calculated (non-ln-transformed data). Element concentrations (ln-transformed) were compared between sites using one-way analysis of variance. Non-ln-transformed data were compared against respective standards/recommended values using the Student’s t test (one-way T). Generalised linear modelling (ANOVA) was used to compare element values between different media and sites. Index of geoaccumulation, enrichment factor, pollution index, average daily dose, hazard quotients, hazard indices and non-cancer and cancer health risks were also calculated.

## Results and discussion

### Elemental concentrations in environmental samples

The results showed relatively high concentrations of PTEs in outdoor soils, ores, mine wastes (Table 1), sediments (Table 3) and water (Table 4) from ASGM villages that varied significantly within sites and across the three media compared to control sites and WHO/CCME/US EPA recommended values and standards, particularly for As, Hg, Cr and Pb.

**Table 1:** Total element concentrations in soil

Sample origin *n*	Industrial (20)	Ores (17)	Mine waste /tailings (25)	Residential (19)	Agricultural (19)	Schools and playgrounds (6)	Exhausted mine waste/tailings (6)	Control residential (3)	Control agricultural (2)	Control playground and school (3)	Control forest (1)	Background values (45)
Element (mg kg^−1^)												
As												
Median	221*	74*	532^*^	34.4^*^	24.4^*^	10.3^*^	538^*^	13.8^*^	7.2	10.8	2	3.49
95th percentile	3502^*^	3154^*^	9745^*^	2070^*^	459*	1218*	3385^*^	14.16^*^	10.71	11.52	2	33.01^*^
US EPA/CCME	12	12	12	12	12	12	12	12	12	12	12	–
Cd												
Median	0.19	0.25	0.16	0.13	0.17	0.17	0.52	0.11	0.17	0.11	0.33	0.08
95th percentile	0.97	1.33	2.18	0.71	0.4	1.18	4.31	0.15	0.40	0.16	0.33	0.26
CCME	10	10	10	10	1.4	10	10	10	1.40	10	10	–
Cr												
Median	216^*^	179^*^	141.7^*^	264.2^*^	197^*^	680.2^*^	195^*^	113.2^*^	172.8^*^	101.8^*^	175^*^	78.9^*^
95th percentile	988^*^	278^*^	789.4^*^	***1351***	626^*^	1560.7^*^	352^*^	210.2^*^	210.5^*^	135.8^*^	175^*^	308.5^*^
CCME	64	64	64	64	64	64.00	64	64	64	64	64	–
Hg												
Median	1.5^*^	0.38	1.55^*^	1.07^*^	0.49	*0.21*	2.58^*^	0.05	0.04	0.03	0.08	n/a
95th percentile	26.5^*^	8.78^*^	68.16^*^	6.34^*^	***17.42***	3.35^*^	6.98^*^	0.05	0.04	0.04	0.08	n/a
US EPA	1	1	1	1	1	1	1	1	1	1	1	–
Ni												
Median	74.2^*^	91.5^*^	50.3^*^	84^*^	66.9^*^	116^*^	57.5^*^	38.6	59.0^*^	28.5	91.4^*^	26.8
95th percentile	246.6^*^	127.4^*^	180.6^*^	291^*^	183.2^*^	426^*^	76.6^*^	85.9^*^	87.9^*^	34.4	91.4^*^	82.6^*^
CCME	50	50	50	50	50	50	50	50	50	50	50	–
Pb												
Median	36.4	37.6	40.5	24.9	21	34.8	104.6	15.4	17.7	18.8	8.5	0.06
95th percentile	196.9	270.5	255.7	152.2	62.2	206.7	186.2	15.7	23.5	34.6	8.5	0.12
US EPA	400	400	400	400	400	400	400	400	400	400	400	–

Values in bold, italics with (*) exceed WHO/CCME/US EPA recommended values/standards. Background values as reported by Watts et al., (2019)

All soil chemical and physical analyses results with site metadata, quality control/assurance data and limits of detection for analyses are fully presented in Supplementary Table 1a. Median, 95th percentile and the guideline values for As, Cd, Cr, Hg, Ni and Pb in soils are summarised in Table 1, while more detailed information for soil categories, chemical parameters and descriptive statistics for all elements is reported in Supplementary Table 2. X-Ray diffraction (XRD) mineral analysis results with site metadata and descriptive statistics for the ores are summarised in Supplementary Table 1b. Bioavailability/bioaccessibility data for agricultural, ores, mining and ore processing sites, playgrounds, schools and residential soils (*n* = 20) (fully presented in Supplementary Table 1c) are summarised in Table 2 for As, Cd, Cr, Hg, Ni and Pb.

**Table 2:** Percentage of gastric bioaccessible elements measured in soil and the bioaccessible hazard quotient for soil ingestion in adults and children

Total (mg kg^−1^) | BAF (%)		As		Cd		Cr		Hg		Ni		Pb	
Sample type (*n* samples)		Total	As%	Total	Cd%	Total	Cr%	Total	Hg%	Total	Ni%	Total	Pb%
Agricultural soils (*2*)	Range	24–103	2–3	0.1–0.3	46–51^*^	102–257	0.3	6.8–21.1	35–51^*^	44–78	6–9	18–3	24–25^*^
	Mean	64	2	0.2	49^*^	180	0.3	19	43	61	7	28.4	24^*^
Ores, mining and processing sites (*5*)	Range	96–5348	1–6	0.1–1	2–42^*^	128–1005	0–18^*^	0.03–88	1–72^*^	16–239	0.4–14	25–290	2–30^*^
	Mean	2272	3	0.6	19^*^	362	4.3	21.2	19^*^	112	6	125	17^*^
Schools, playgrounds and residential soils (*13*)	Range	4.8–8567	2–60^*^	0.06–1.03	11–56^*^	73–1682	0.1–1 2	0.07–6.9	2–39^*^	12–445	4–10	18–181	1–35^*^
	Mean	640	9	0.27	35^*^	640	1.8	2.1	10	166	7	47	19^*^
BHQ (adults)	Range	0.04 –289^*^		0.004–0.055		0.05–1.57		0.01–4.95*		0.003–0.15		0.01–1.31*	
	Mean	***26.8***		0.021		0.19		0.81		0.05		0.37	
BHQ (children)	Range	0.4–2700^*^		0.04–0.51		0.05–14.6*		0.1–46.2*		0.03–1.06*		0.13–1.22*	
	Mean	20.5*		0.16		0.79		0.92		0.27		2.4*	

BAF values in bold, italics with (*) exceed 15% and are considered high. BHQ values in bold, italics with (*) exceed 1 and indicate health risks associated with ingestion of PTE-polluted soils

BAF%, bioaccessible fraction%; BHQ, bioaccessible hazard quotient

Sediment physical and chemical analyses data with site metadata, quality control/assurance data and limits of detection for analyses results are broadly presented in Supplementary Table 3. The spatial distribution and variation, median, 95th percentile and the respective guideline values for As, Cd, Cr, Hg, Ni and Pb in sediments from streams and rivers, springs, washing ponds, exhausted mine tailings and fishponds within ASGM and non-ASGM villages in Kakamega and Vihiga counties are shown in Table 3, while the sediment categories, chemical parameters and descriptive statistics for all elements are presented fully in Supplementary Table 4.

**Table 3:** Elemental concentrations in sediment

Parameter		Sediment element concentrations (mg kg^−1^)					
Sample origin (*n*)		Washing ponds (17)	Fishponds (2)	Stream/river (6)	Exhausted mine tailings (2)	Spring (6)	Control river (1)
As	Median	345^*^	10.1^*^	62.4^*^	906.9^*^	35.1^*^	6.2
	95th percentile	2680^*^	14^*^	155^*^	975^*^	69^*^	6
	European Union Standard	20	10	20	20	20	20
Cd	Median	0.17	0.1	0.08	0.99	0.06	0.13
	95th percentile	0.66	0.11	0.15	0.9	0.11	0.13
	European Union Standard	2.3	2.3	2.3	1.38	2.3	2.3
Cr	Median	228^*^	582^*^	213^*^	195^*^	198^*^	613^*^
	95th percentile	972^*^	1042^*^	928^*^	231^*^	209^*^	613^*^
	European Union Standard	81	81	81	81	81	81
Hg	Median	6.1^*^	2.4^*^	3.9^*^	2.39^*^	7.2^*^	< 0.02
	95th percentile	10.3^*^	2.5^*^	6.9^*^	3.46^*^	12.6^*^	< 0.02
	European Union Standard	0.15	0.15	0.15	0.15	0.15	0.15
Ni	Median	102*	203*	77*	33*	24*	95*
	95th percentile	190*	364*	184*	42*	58*	95*
	European Union Standard	21	21	21	21	21	21
Pb	Median	20.1	14.6	17.95	387^*^	17.7	15.8
	95th percentile	154.5	15.32	40.48	669^*^	35.9	15.8
	European Union Standard	120	120	120	120	120	120

Values in bold, italics with (*) exceed European Union Standards for elements in sediment (Tornero et al., 2019)

Drinking and non-drinking water samples, their physical and chemical analyses data, location, source and usage information is fully presented in Supplementary Table 5. Descriptive statistics for all measured parameters in both drinking and non-drinking water from ASGM and non-ASGM are summarised in Table 4, but fully presented in Supplementary Table 6. The water quality criteria for this study were based on the World Health Organization guidelines (WHO, 2003, 2006, 2017, 2021).

**Table 4:** Element concentrations in water

Sample origin (*n*)	Drinking water in ASGM (24)	Drinking water in non-ASGM (5)	Non-drinking water sources in ASGM	
			Washing ponds **(**11**)**	Fishponds **(**2**)**
As				
Median	2.33	0.65	7.75	2.01
95th percentile	18^*^	1	468^*^	3
WHO guideline value	10	10	20	20
Cd				
Median	0.05	0.01	0.04	0.03
95th percentile	0.1	0.03	1.22	0.04
WHO guideline value	3	3	10	0.2
Cr				
Median	0.48	0.3	0.50	1.54
95th percentile	2.73	1.42	2.66	2.81
WHO guideline value	50	50	4.9	50
Pb				
Median	0.18	0.1	0.2	0.25
95th percentile	0.39	0.26	0.47	0.32
WHO guideline value	10	10	200	200
Ni				
Median	1.57	0.64	4.81	3.60
95th percentile	10	14	99	6
WHO guideline value	20	20	70	70

Values in bold, italics with (*) exceed WHO recommended values

World Health Organization (WHO) guidelines for drinking water, recreational water and water sources (WHO, 2003, 2006, 2017, 2021)

The results of PI and NIPI (≥ 3) showed that the sampled soil, sediment and water were highly polluted with the PTEs. Generated EF, Igeo, PI and NIPI values for As, Cd, Cr, Hg, Ni and Pb in various categories of soil, sediment and water are fully presented in Supplementary Table 9. According to the Muller Scale (Förstner & Müller, 1981), the calculated Igeo results indicated that As was of most concern for soil, sediment and water matrices in this study. The decreasing order of contribution of various PTEs studied on the basis of Igeo follows: As > Cr > Hg > Ni > Pb > Cd (Supplementary Table 9), and each elemental result is further discussed.

#### Arsenic

Exposure to As has been reported to cause: cancer; significant adverse impacts on neurodevelopment; behavioural disorders; cardiovascular and respiratory problems; skin lesions; and anaemia during pregnancy (Duker et al., 2004, 2006; IARC, 2012). Extremely high As concentrations were found at the mining and ore processing sites (ores, industrial sites, mine wastes/tailings and exhausted mine waste/tailings) with 96% of soil samples having an As concentration (Tables 1, 2, 3, 4; Supplementary Tables 1–6) up to 7937 times greater than the 12 mg kg^**−**1^ US EPA and CCME 12 mg kg^**−**1^ standard for residential soils. Similarly, with a high of up to 8567 mg/kg As, 64% of outdoor soils from residential areas, schools, playgrounds and agricultural areas exceeded the standard (Table 1a; Supplementary Table 2). The soil As concentrations (mean As, 1652 mg kg^−1^; range 1.4–95,239 mg kg^−1^) found in this study are significantly higher than those previously reported in ASGM areas in Migori Gold Belt and Transmara, Kenya, by Odumo et al. (2018) (mean As, 546; range 2.1–17,250 mg kg^−1^) and Ngure et al. (2014) (As 0.08–86 mg kg^−1^). The previous studies, however, lack laboratory data transparency including uncertainty measurements. Sediment samples (*n* = 36) similarly recorded high As concentrations with 67% exceeding the 20 mg kg^−1^ EU standard (Table 3; Supplementary Table 3). Water from ore washing ponds (*n* = 11) in ASGM villages had As concentrations ten times higher than the WHO 20 µg L^−1^ guideline, while drinking water from shallow wells and boreholes had As concentrations up to two times higher than the WHO recommended 10 µg L^−1^ limit for drinking water in 25% of the samples (WHO, 2021) (Table 4; Supplementary Table 6).

#### Cadmium and lead

The study found Cd and Pb concentrations within respective guidelines in soils, except for exhausted mine waste/tailings sediments from cyanidation plants (Tables 1, 2, 3) and a single sample from a mine waste/tailing heap that had 14.5 mg kg^−1^ and 6,454 mg kg^−1^, respectively. Cadmium and Pb concentrations did not exceed the guideline value for water as well. However, Cd and Pb are extremely toxic and accumulate in the body, even low-level chronic exposures are hazardous over time (WHO, 2010a). Cadmium is a human carcinogen (Group 1) and affects the kidney, skeletal and respiratory systems (Faroon et al., 2013; IARC, 2021; WHO, 2010b). Exposure to Pb can cause permanent adverse effects on brain development, and similar to Cd, kidneys, the blood, nervous and skeletal systems in children (Abadin et al., 2007; Cecil et al., 2008; Chakrabarty et al., 2014; Mazumdar et al., 2012). Childhood Pb exposure was also reported to alter gene expression and increase the risk of disease and death later in life. These Cd and Pb results corroborate the previous study findings in the Migori Gold Belt and Transmara, Kenya, except for ores from Makalder, Migori (*n* = 15; mean Pb, 1,048 mg kg^−1^; range 3.1–11,149 mg kg^−1^) (Odumo et al., 2018).

#### Chromium and nickel

The presence of elevated Cr and Ni in soil and sediment samples both in the ASGM, the control and background samples (Tables 1, 3) may indicate geogenic sources and presence of other routes of dispersion for Cr and Ni other than ASGM activities. Chromium concentrations in 98% of all the soil samples and 75% of all the sediment samples exceeded the respective CCME guideline value (64 mg kg^−1^), 26 times higher for soils and the 81 mg kg^−1^ EU standard for Cr in sediments. Concentrations of Ni in 70% (85 out of 121) soil and 78% (28 out of 36) sediment samples from ASGM exceeded respective CCME Guideline values and European Union standards. Additionally, the results also revealed high Ni concentrations (95th percentile; 25.6 µg L^−1^ and, range 0.9–32 µg L^−1^) in drinking water sources (*n* = 24) and washing ponds (*n* = 11) (mean; 26 µg/L, range 2.2–218 µg L^−1^) above the respective WHO guideline values (WHO, 2021) (Table 4; Supplementary Table 6).

Chromium and its compounds cause various health problems including: lung cancer, kidney and liver damage, alteration of genetic material, skin rashes, stomachs upset and ulcers, respiratory problems, weakened immune systems, nasal irritation, nasal ulcer and hypersensitivity reactions such as contact dermatitis, asthma and death (Hamilton et al., 2018; IARC, 2021; Kaczmarek et al., 2007). Nickel exposure has been reported to cause lung cancer; nasal cancer; gastrointestinal manifestations; respiratory manifestations; lung fibrosis; contact dermatitis; headaches; cardiovascular diseases; and epigenetic effects (Genchi et al., 2020).

#### Mercury

Elevated Hg concentrations were found in soils in ASGM villages. Concentrations of Hg in 49% of the samples were up to 144 times greater than the US EPA 1 mg kg^−1^ limit for residential soils (Table 1: Supplementary Tables 1a and 2). All sediment samples (100%) from washing ponds, fishponds, streams, rivers, exhausted mine wastes and springs had Hg concentrations above the 0.15 mg kg^−1^ European Union standard (Supplementary Table 3). Previous studies Hg concentrations than this study’s in ASGM areas in Migori Gold Belt and Transmara, Kenya (0.01–31.5 mg kg^−1^), Niger, Nigeria (0.002–20.99 mg kg^−1^) (Odukoya et al., 2021), and Zamfara, Nigeria (0.01–68.1 mg kg^−1^) (Plumlee et al., 2013). Ore processors introduce Hg into the mining environment during the amalgamation process for recovering gold and dumping of mine tailings. Mercury is removed from the amalgam by burning during which Hg vaporises into the atmosphere. The Hg vapour can be directly inhaled by humans and animals or may fall as droplets in the nearby soils, sediments and waters. Mercury is a powerful neurotoxin and affects the digestive and immune systems, lungs, kidneys, skeleton, skin and eyes (Risher, 1999). Inhalation of Hg vapour poses the greatest risk to the ASGM workers and residents. The lungs readily absorb the gaseous Hg particles into the blood stream and circulate them throughout the body. Thus, more Hg reaches the brain and may result in permanent profound damage to the brain, kidneys, developing foetuses and other vital organs and systems (Bose-O'Reilly et al., 2010).

### Bioaccessibility and bioacccessible hazard quotient

The bioaccessibility results reflected the amounts of PTEs that could be absorbed into human bodies through the gastrointestinal tract (GIT) following incidental ingestion of soils in ore mining and processing areas, agricultural farms, schools, playgrounds and residential areas (Supplementary Table 1c: (i); Table 2). High bioaccessibility of a given PTE indicates a high potential for it to be absorbed into the body. The means and ranges of bioaccessible As, Cd, Cr, Hg, Ni and Pb in the stomach alone and combined stomach + intestines varied significantly as given in Supplementary Table 1c: (i). The gastric BAF% mean and range values for As, Cd, Cr, Hg, Ni and Pb summarised in Table 2 depict the forms of the PTEs in the soils which make them to either be mobile and become readily absorbed in the GIT (BAF% above 15%) or not (BAF below 15%), following incidental ingestion by children and adults. Mercury (35–51%) and Pb (24–25%) in agricultural soils; Hg (1–72%) in ore mining and processing sites; and Hg (2–39%) and As (2–60%) in schools, playgrounds and residential soils showed the highest bioaccessibility (Table 2).

Using the total PTE concentration alone in assessing health risk in soils may lead to over estimation of the actual risk posed because only a proportion of a PTE is usually absorbed (Odukoya et al., 2021). Combining gastric bioaccessibility and the health risk assessment will help in implementing a more reasonable risk management of PTEs in ASGM. For this study, the bioaccessible hazard quotients (BHQ) (Table 2: Supplementary Table 1c: (iii)) were also calculated for soil ingestion pathway for both children and adults. For children, BHQ values in 90% of the soils for As (range 1.1–2701) and Pb (range 1.2–12.2); 40% and 30% of the soils for Cr (range 1.03 –14.6) and Hg (range 3.8–46.2), respectively, exceeded the safe limit (BHQ ≤ 1). However, for adults, BHQ values in 55%, 5%, 15% and 10% of the soils for As (range 1.9–289), Cr (range 1.6), Hg (range 2.6–6) and Pb (range 1.2–1.3) exceeded the safe (BHQ ≤ 1) limit, indicating a lower health risk for adults compared to children (Supplementary Table 1c: (iii)). The high daily soil ingestion levels, soil ingestion hazard quotients, gastric bioaccessible fractions (up to 72%) and the elevated bioaccessible hazard quotients found in this study indicated plausibly high soil dust ingestion rates that could cause problematic PTEs uptake likely to damage the health of ASGM workers and local communities (Supplementary Table 1c: Table 2). This study’s results corroborate with previous studies in ASGM areas in Nigeria, although the BHQ values found in this study are much greater than those previously found (0.000005–4.04) (Odukoya et al., 2021).

### Ore mineral characterisation

As previously reported, the gold found in Kakamega and Vihiga counties is mainly associated with quartz veins (quartz-carbonate and quartz-vanadium mica veinlets) and sulphide mineralisation (pyrite, pyrrhotite, sphalerite, arsenopyrite, galena and molybdenite) in Precambrian gold-bearing greenstone rocks (Kavirondian, Nyanzian, granites and basalts) (Huddleston, 1954; Ichang & MacLean, 1991; MoA, 2009; Pulfrey, 1969). Similar to other greenstone gold mining regions, the gold-bearing ores studied from Kakamega and Vihiga counties were predominantly composed of quartz (up to 72%), feldspar (up to 45%) and mica (up to 22%) with a small proportion of richterite (up to 5%), clinochrysotile (up to 2%), chlorite (up to 5%), calcite (up to 3%), pyrite (1%) and hematite (1%) (Supplementary Table 1b).

From elemental ore data (Table 1) and the mineral characterisation data (Supplementary Table 1b), it is evident that the geology of ore deposits, crude crushing and mechanised grinding of ores in ASGM are the fundamental causes of the high As, Pb, Hg, Cr, Cd and Ni concentrations in soils, sediments and water in the ASGM villages in Kakamega and Vihiga counties. Enrichment factor values indicated enrichment and pollution of soils, sediments and water with PTEs from anthropogenic activities mainly artisanal small-scale gold mining and ore processing (Supplementary Table 9). The As, Pb and Cr indicate the existence of arsenopyrite (FeAsS), galena (PbS) and chromite [(Fe, Mg, Al) Cr_2_O_4_] ore veins, respectively. Nickel exists in gold-bearing ores as pentlandite (FeNi)S_8_ and pyrrhotite (Fe_(1−*X*)_S), which may contain up to 5% Ni in some ores. Cadmium occurs in the form of an isometric trace element in sphalerite ore veins in varying concentrations. In addition to introduced Hg by ore processors including amalgam burners, as revealed in this study, the mine wastes/tailings which is often disposed of directly in nearby soils in farms and homes and river channels constitute potential environmental risk as secondary sources of the high As, Cd, Cr, Hg, Ni and Pb concentrations found. Surface runoffs may also wash PTEs from the ASGM activities and sites into surface and ground water sources explaining the high PTEs concentrations found in the sediments.

Arsenic, Cd, Cr, Hg, Ni and Pb are extremely toxic to human health (Abadin et al., 2007; Chou & Harper, 2007; Risher, 1999). Several reports linked As, Pb, Hg, Cr, Cd and Ni among other pollutants from ASGM to several deaths and various non-communicable diseases such as cancer, compromised immunity, stroke, neurodevelopmental disorders, asthma, birth defects in childhood, liver, lung, heart and kidney diseases, blood disorders and skeleton disorders among others in children and adults (Dooyema et al., 2012; Laborde et al., 2014; Landrigan et al., 2015; Plumlee et al., 2013; Steckling et al., 2014, 2017). Susceptible groups are persons that are more sensitive toxic effects of these elements (As, Cd, Cr, Hg, Ni and Pb) and their compounds. These include sick individuals, the foetus, the new-born, and children, and persons exposed to higher dosages like the amalgam burners in ASGM (Abadin et al., 2007; Chou & Harper, 2007; Fashola et al., 2016; Risher, 1999).

### Estimated potential human exposure and health risk

The study quantified both potential cancer and non-cancer health risks among adults and children living or working in ASGM in the studied villages in Kakamega and Vihiga Counties.

#### Non-cancer health risk of toxic elements for adults and children

**Fig. 2:**
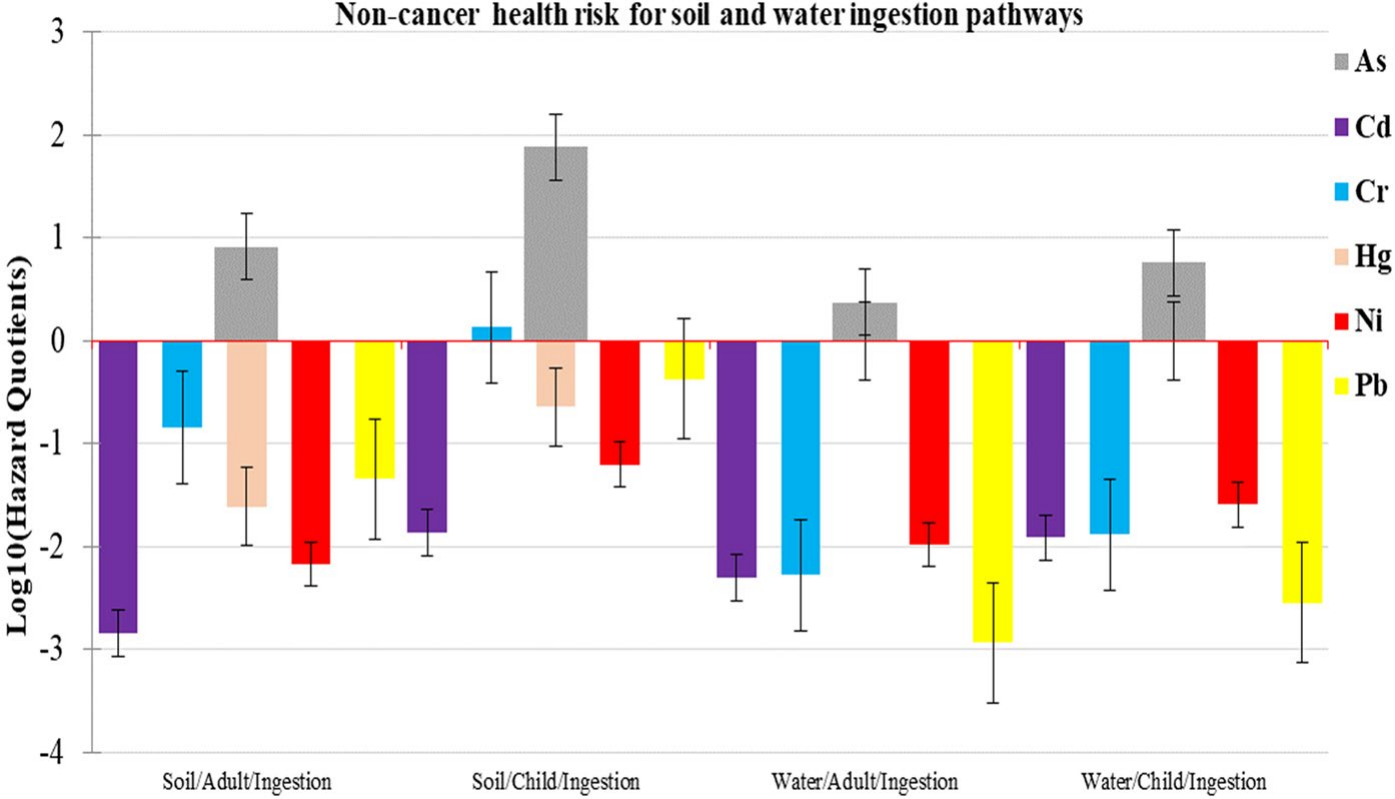
Non-cancer health risk results for soil and water ingestion in adults and children

**Fig. 3:**
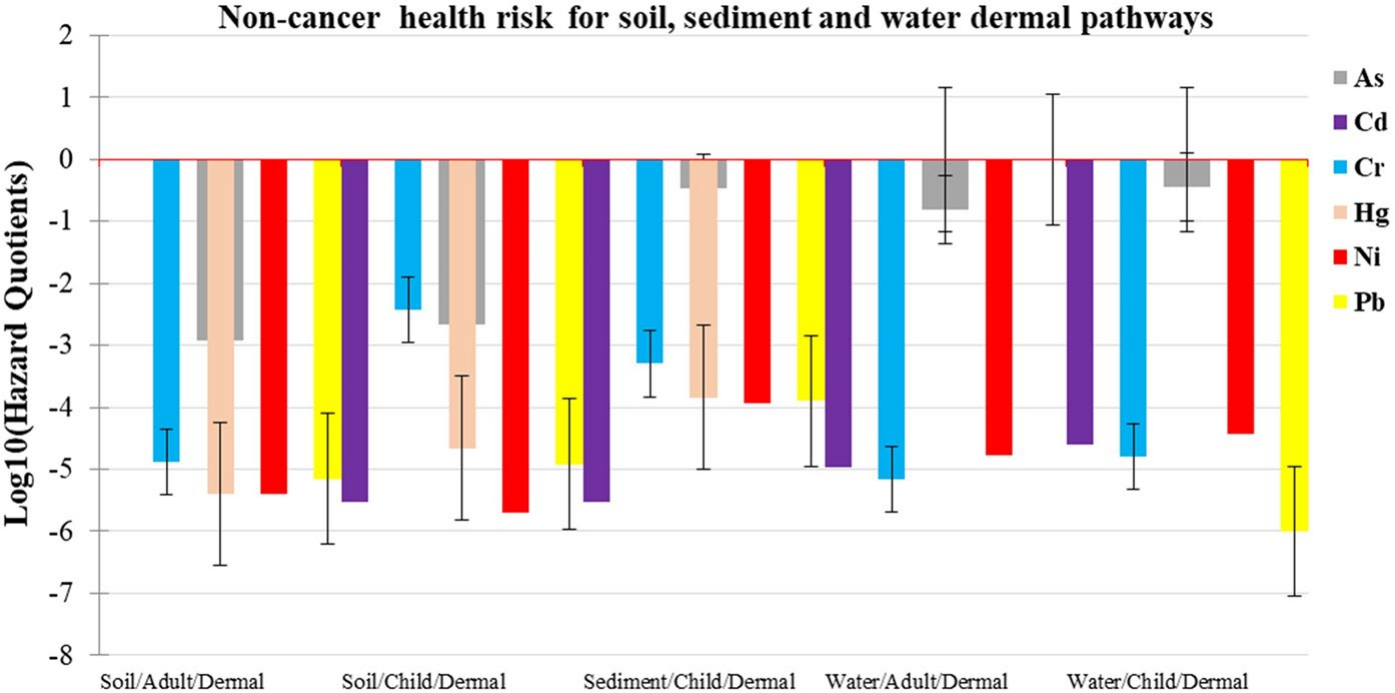
Non-cancer health risk in adults and children for soil, sediment and water dermal pathway

**Fig. 4:**
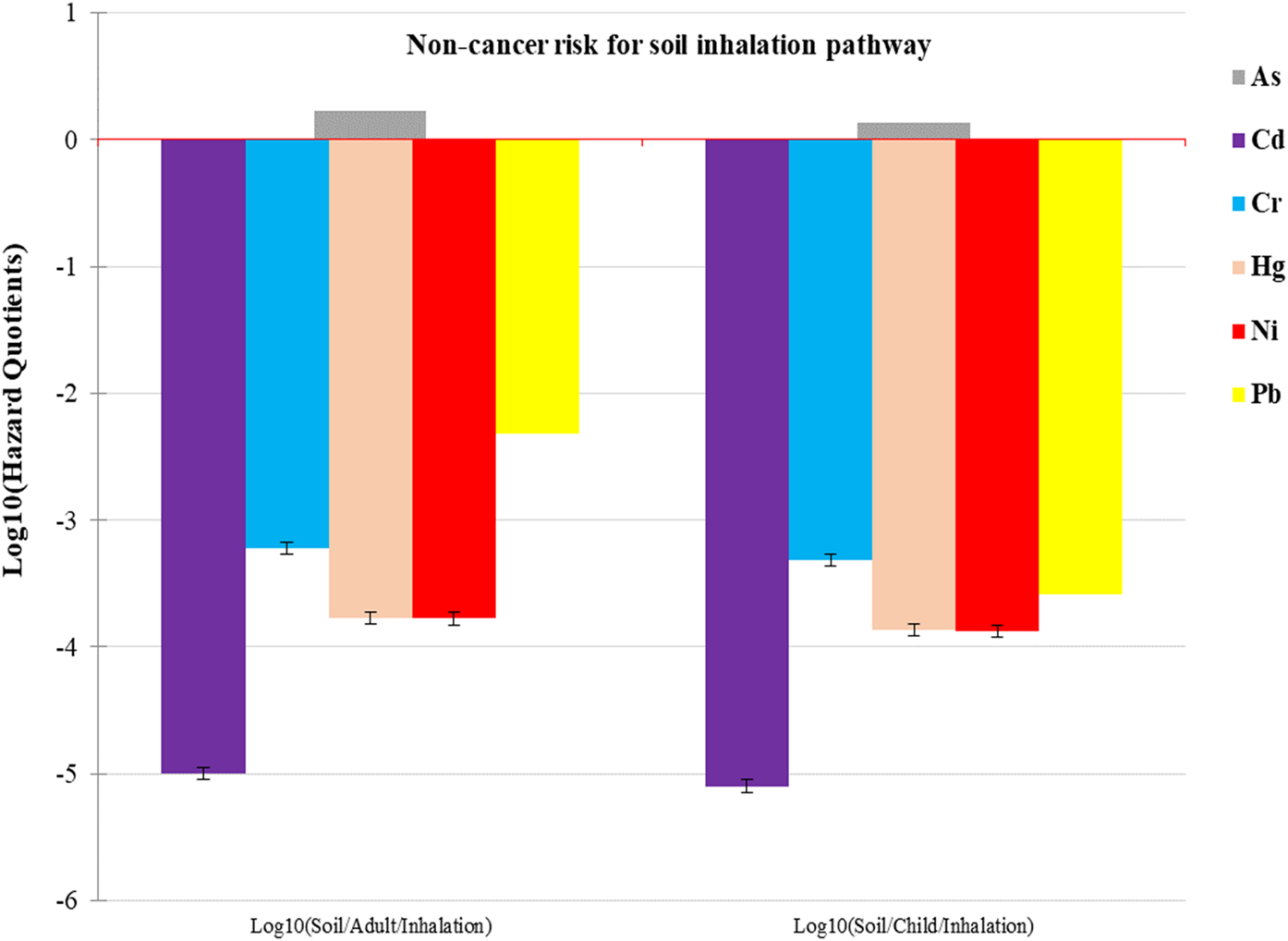
Non-cancer health risk results for soil inhalation in adults and children

The calculated risk of adverse non-cancer health effects for both adult humans and children, based on RfD values (Supplementary Table 8) and the estimated average daily doses (ADD) (Supplementary Table 10a) is given as hazard quotients (HQ), fully presented in Supplementary Table 10b. These non-cancer health risk findings are further summarised for the ingestion, dermal contact and inhalation pathways in Figs. 2, 3 and 4 in terms of hazard quotients (HQs). The hazard index (HI) values for adults, presented in Supplementary Table 10c, and children (Supplementary Table 10d) sum up the total non-cancer risk per pathway in a particular setup.

Values of HQ, HI and HIt less than 1 indicate no obvious risk to the population. But if these values are greater than 1, there may be concern for potential non-cancer health effects (Gerba, 2019; Means, 1989; USEPA, 2004). For the adult population, calculated values of HQ were greater than one in soil ingestion (8.15), soil inhalation (1.7) and water ingestion (2.34) pathways for As (Figs. 2, 3, 4). Similarly, the mean HI values for ingestion (10.5), inhalation (1.7) pathways and the 75th percentile HI values for ingestion (3.69) and dermal contact (1.01) pathways were greater than 1 for As (Supplementary Table 10 c). The mean HIt value (12.4) and the 75th percentile HIt value (4.7) exceeded 1 as well. These meant that the adult population in ASGM villages within Kakamega and Vihiga counties was at risk of non-cancer effects from As exposure. For children, HQ values for soil ingestion (76), soil inhalation (1.36) and water ingestion (5.72) pathways exceeded 1 (Figs. 2, 3, 4) for As and Cr. Values for HI Ingestion (mean HI = 81.7; HI at 25th percentile = 1.03; median HI = 2.96; HI at 75th percentile = 26.9 for As; and mean HI = 1.36; HI at 75th percentile = 1.27 for Cr) and inhalation (mean HI = 1.36 for As) also exceeded 1 (Supplementary Table 10d).

Furthermore, the HIt values for all pathways mainly driven by As and Cr were greater than 1. The total mean non-cancer health risk for both adults and children was found to be 98.6. The total non-cancer risk was found to be 3.45 at the 75th percentile; 4.92 at the 50th percentile; and 1.99 at the 25th percentile for all the pathways investigated. These high values indicate toxic element pollution that may pose an extremely high non-cancer health risk to people living in the ASGM villages in Kakamega and Vihiga counties. The findings also indicate that in both adults and children, ingestion pathway contributes the greatest to non-cancer health risk, followed by the inhalation pathway. Dermal contact is the least contributor to the risk. Similar results were previously reported in gold mining areas of Ogun State, Nigeria (average HQ values: As 7.07 × 10^−2^; Cd 2.34 × 10^−3^; and Hg 7.76 × 10^−4^) (Olujimi et al., 2015), and Krugersdorp, South Africa (HQ values: As, 5.90 × 10^−2^; Cd 5.10 × 10^−1^ and Hg, 3.40 × 10^−2^) (Fashola et al., 2016), with a high non-cancer risk but lower than this study.

#### Cancer risk of toxic elements for adults and children

**Fig. 5:**
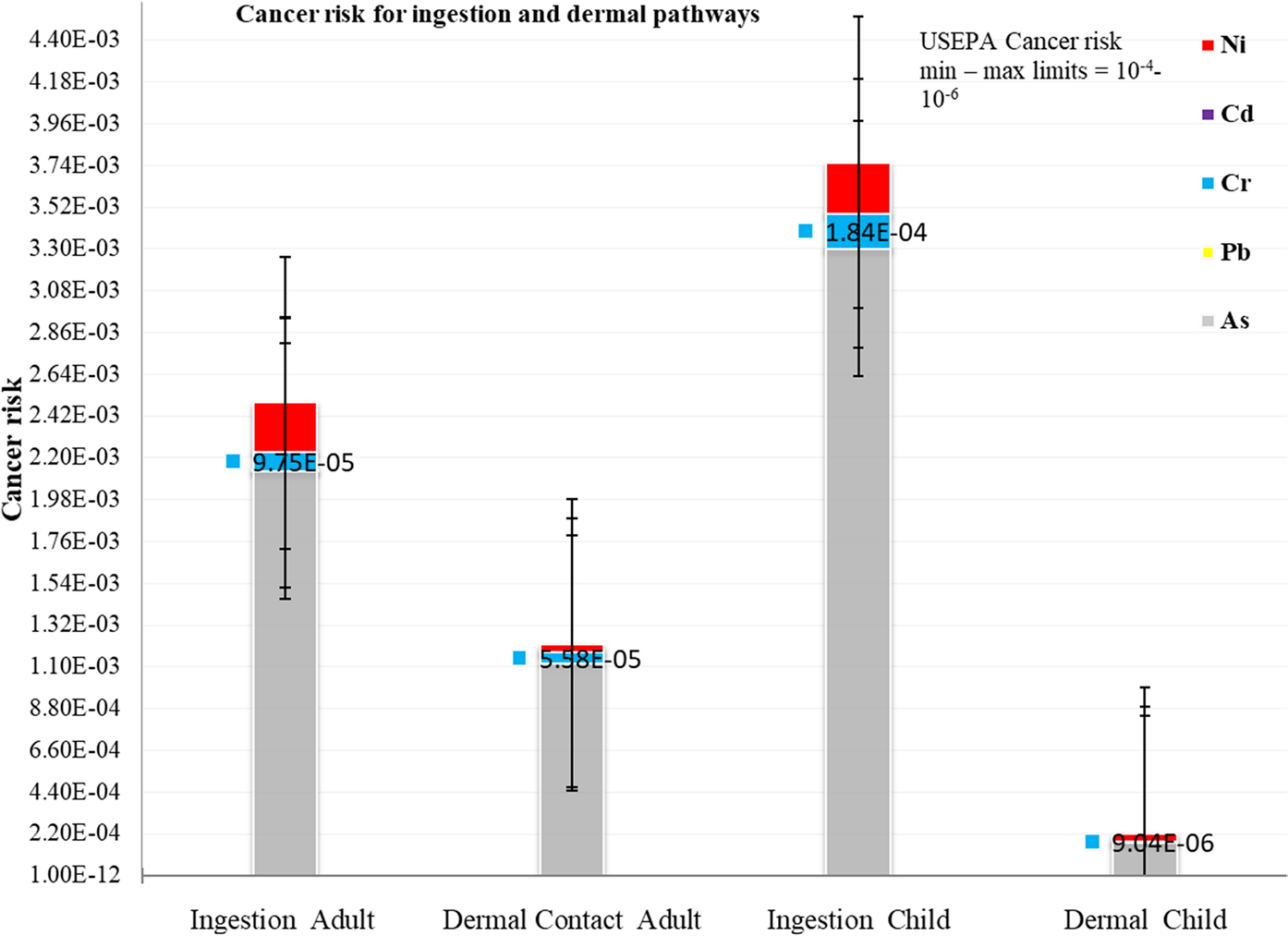
Cancer risk values of toxic elements in adults and children from soil, sediment and water ingestion and dermal contact in ASGM villages in Kakamega and Vihiga counties

**Fig. 6:**
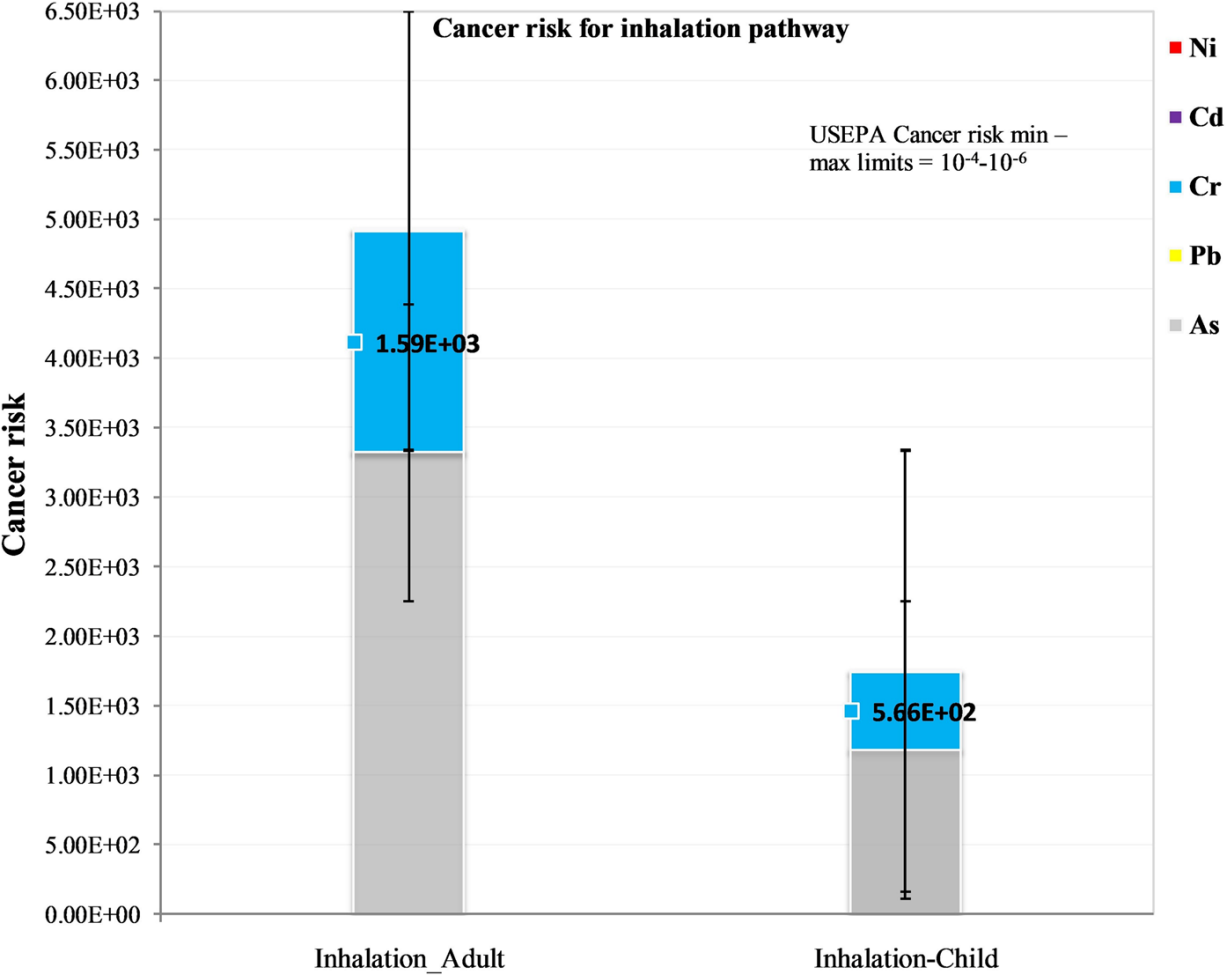
Cancer risk values of toxic elements in adults and children from soil inhalation in ASGM Villages in Kakamega and Vihiga counties

The incremental lifetime cancer risk for adult humans and children was calculated from the respective average contribution of individual toxic elements (As, Cd, Cr, Hg, Ni and Pb) in soil, sediment and water for all the pathways using Eq. 11. The lifetime average daily dose (LADD) values were generated and are fully presented in Supplementary Table 11a. Based on the cancer risk, the lifetime average daily dose (LADD) (Supplementary Table 11a) and the respective cancer potency factors (SF) values (Supplementary Table 8), the incremental lifetime cancer risk (ILCR) values were generated and are summarised in Figs. 5 and 6, but fully presented in Supplementary Table 11b.

The cancer risk was calculated for As, Cd, Cr, Ni and Pb. Mercury is not classifiable as carcinogenic to humans by the International Agency for Research on Cancer (IARC) under the World Health Organization (WHO) (IARC, 2021). Findings revealed that As and Cr were the greatest contributors to the cancer risk (Figs. 5, 6; Supplementary Table 11a). The US EPA guideline values for incremental lifetime cancer risk range between 10^−6^ and 10^−4^. Cancer risk values beyond these are considered an unacceptable risk for both single and multi-element carcinogens (Gerba, 2019; Means, 1989; USEPA, 2004). There are no local cancer risk limits for regulatory decision in Kenya. The cancer risk for individual adults was found to be 4930 and 1750 for children. This means that there is an increased risk of 4930 cancer cases per 100,000 adults in the studied ASGM villages. And, in every 10,000 similarly exposed children below six years, there is an increased risk of 1750 cancer cases. These values were significantly higher than US EPA upper regulatory cancer risk values and are unacceptable, with children being more at risk than adults. The inhalation route is the major contributor to incremental lifetime cancer risk followed by the ingestion pathway.

The results also indicated that in both adults and children, the ingestion pathway was the greatest contributor to the non-cancer health risk followed by the inhalation pathway. For the cancer health effects, the inhalation pathway contributed the most to cancer risk followed by the ingestion pathway. The dermal pathway on the other hand was the least contributor to both non-cancer and cancer risks. As and Cr seemed to contribute the greatest to both cancer and non-cancer risks. This study’s results corroborate previous studies in gold mining areas that reported similarly high cancer risks in Krugersdorp, South Africa (cancer risk 2.2 × 10^−3^) (Fashola et al., 2016) and Nigeria (cancer risk 9.46 × 10^−8^) (Olujimi et al., 2015). The cancer risks in this study exceed those reported in Nigeria and South Africa.

## Conclusion

The soil, sediment and water sources within the ASGM villages in Kakamega and Vihiga counties are highly polluted by potentially toxic elements, especially As, Hg, Cr, Pb and Ni. Soil concentrations of As, Cr and Ni in the studied ASGM villages were 154 (for As), 9 (for Cr) and 4 (for Ni) times higher than the respective background concentrations previously reported by Watts et al. (2019). Community drinking water in the ASGM villages was polluted with As twice the WHO recommended value for As in drinking water. The human health risk assessment results revealed a high risk of cancer among adults (4.93 × 10^−2^) and children (1.75 × 10^−1^) in addition to significant potential non-cancer health effects (risk of 98.6) as a result of exposure to toxic elements in the studied ASGM villages. In both adults and children, ingestion pathway was the greatest contributor to the non-cancer health risk followed by the inhalation pathway. For the cancer health effects, inhalation pathway contributed the most to cancer risk followed by the ingestion pathway. Dermal pathway on the other hand was the lowest contributor to both non-cancer and cancer risks. Arsenic and Cr contributed the greatest risks. There is a potential for ASGM activities to harm the health of ASGM workers who are occupationally exposed and inadvertently harm the health of surrounding inhabitants who are not aware, especially children, who live, play and school near the ASGM operations.

The risk findings in this study are of great environmental/public health concern and call for more in-depth investigations on the possible health effects of ASGM operations on ASGM workers and nearby residents. This study provides critical quantitative evidence of hazards associated with ASGM and will be valuable to the public health and environment management authorities in informing mitigation actions, for example, moving residential areas, farms, children’s playgrounds and schools away from ASGM activities; control of dust transfers; replacing toxic materials used in ASGM such as Hg and CN with non-toxic alternatives; providing alternative safe drinking water sources to the ASGM workers and residents; and cleaner technological interventions in ore excavation, ore processing and Au recovery; industrial hygiene; and public health policy formulation in relation to ASGM in Kenya.

## Supplementary Information

Below is the link to the electronic supplementary material.

Supplementary file1 (XLSX 54457 KB)

## Data Availability

All the data are readily available in supplementary tables 1–12.
